# Evidence of validity of the Smoking Cessation Counseling scale - Brazilian version

**DOI:** 10.1590/1518-8345.6587.4125

**Published:** 2024-03-15

**Authors:** Juliana Maria Ruoco Zambardi Porreca, Robin Purdy Newhouse, Vinicius Batista Santos, Juliana de Lima Lopes, Alba Lúcia Bottura Leite de Barros

**Affiliations:** ^1^ Universidade Federal de São Paulo, Escola Paulista de Enfermagem, São Paulo, SP, Brazil; ^2^ Indiana University, School of Nursing, Indiana, IN, United States of America.

**Keywords:** Counseling, Methodological Study, Public Health Nursing, Cessation Tobacco, Validation Study, Factor Analysis Statistical, Consejo, Estudio Metodológico, Enfermería en Salud Pública, Cese del Tabaquismo, Estudio de Validación, Análisis Factorial, Aconselhamento, Estudo Metodológico, Enfermagem em Saúde Pública, Abandono do Tabagismo, Estudo de Validação, Análise Fatorial

## Abstract

**Objective:**

to evaluate the evidence of validity of the internal structure and reliability of the Brazilian version of the Smoking Cessation Counseling instrument

**Method:**

psychometric study of confirmatory factor analysis and reliability carried out on 250 nurses in clinical practice. For the analysis of the convergent validity of the factor model, Average Variance Extracted values were calculated, and discriminant analysis was carried out using the Fornell-Larcker criterion. Reliability was examined using Cronbach’s alpha coefficient and composite reliability

**Results:**

it was necessary to exclude seven items from the Advanced Counseling domain and one item from the Basic Counseling domain in order to properly obtain the Average Variance Extracted values and the Fornell-Larcker criterion. The composite reliability ranged from 0.76 to 0.86 and the overall Cronbach`s alpha coefficient was 0.86, ranging from 0.53 to 0.84 depending on the domain assessed. The final version of the instrument was made up of 16 items divided into 4 domains

**Conclusion:**

the Brazilian version of Smoking Cessation Counseling obtained adequate psychometric evidence of validity and reliability. Further studies are needed to refine the instrument.

## Resumen

**Objetivo:**

evaluar las evidencias de validez de la estructura interna y de la confiabilidad de la versión brasileña del instrumento Smoking Cessation Counseling.

**Método:**

estudio psicométrico de análisis factorial confirmatorio y de confiabilidad realizado en 250 enfermeras de la práctica clínica. Para el análisis de la validez convergente del modelo factorial se calcularon valores de Average Variance Extracted, el análisis discriminante se realizó mediante el criterio de Fornell-Larcker. La confiabilidad se examinó por el coeficiente de alfa de Cronbach y por la confiabilidad compuesta.

**Resultados:**

fue necesaria la exclusión de siete ítems del dominio de Asesoramiento avanzado y un ítem del dominio Asesoramiento básico para obtener adecuadamente los valores de Average Variance Extracted y del criterio de Fornell-Larcker. La confiabilidad compuesta varió de 0,76 a 0,86 y el coeficiente de alfa de Cronbach global alcanzado fue de 0,86, variando de 0,53 a 0,84 dependiendo del dominio evaluado. Se obtuvo la versión final del instrumento compuesto de 16 ítems distribuidos en 4 dominios.

**Conclusión:**

la versión brasileña de Smoking Cessation Counseling obtuvo adecuadas evidencias psicométricas de validez y confiabilidad. Estudios posteriores serán necesarios para el refinamiento del instrumento.

## Resumo

**Objetivo:**

avaliar as evidências de validade da estrutura interna e da confibialidade da versão brasileira do instrumento *Smoking Cessation Counseling*

**Método:**

estudo psicométrico de análise fatorial confirmatória e de confiabilidade realizado em 250 enfermeiras da prática clínica. Para a análise da validade convergente do modelo fatorial foram calculados valores de *Average Variance Extracted* , a análise discriminante foi realizada pelo critério de Fornell-Larcker. A confiabilidade foi examinada pelo coeficiente de alfa de Cronbach e pela confiabilidade composta

**Resultados:**

foi necessária a exclusão de sete itens do domínio de Aconselhamento avançado e um item do domínio Aconselhamento básico para obtenção adequada dos valores de *Average Variance Extracted* e do critério de Fornell-Larcker. A confiabilidade composta variou de 0,76 a 0,86 e o coeficiente de alfa de Cronbach global alcançado foi de 0,86, variando de 0,53 a 0,84 a depender do domínio avaliado. Obteve-se a versão final do instrumento composto de 16 itens distribuídos em quatro domínios

**Conclusão:**

a versão brasileira da *Smoking Cessation Counseling* obteve adequadas evidências psicométricas de validade e de confiabilidade. Estudos posteriores serão necessários para o refinamento do instrumento.

**Highlights**

**(1)** Instrument shows adequate internal consistency and validity

**(2)** Evaluate counseling practices and their impact on patient care

**(3)** Instrument for evaluating smoking cessation counseling

**(4)** Provides important information for planning nursing care.

## Introduction

 Smoking is considered by the World Health Organization (WHO) to be the leading cause of preventable death worldwide. It is estimated that one third of the world’s population are smokers, or 1.2 billion people ^(^ ^1^ ^)^ . Around 8 million people died from tobacco-related illnesses in 2017. It is believed that the number of annual deaths is likely to rise even after tobacco use rates begin to fall, because diseases related to this addiction can manifest themselves late in life ^(^ ^2^ ^)^ . 

 In Brazil, there has been a substantial and effective effort on the part of health professionals to formulate public policies and implement tobacco control measures ^(^ ^3^ ^)^ . In 2005, Brazil became a signatory to the World Health Organization’s Framework Convention on Tobacco Control (WHO-FCTC), whose guidelines form the basis of the country’s National Tobacco Control Policy (NTCP). The results of implementing the NTCP have translated into a notable reduction in the prevalence of smokers and in the morbidity and mortality associated with tobacco consumption ^(^ ^4^ ^-^ ^5^ ^)^ . 

 The professional teams working in the NTCP are made up of different professionals, whose activities depend on their category, including physicians, nurses, psychologists, social workers, nutritionists, physiotherapists and nursing technicians ^(^ ^5^ ^-^ ^6^ ^)^ . 

 As an integral part of the multidisciplinary team in healthcare facilities, nurses play a crucial role in identifying areas and modalities of action, which in turn contributes to defining and guiding their professional practice. In this scenario, nurses’ responsibilities include the prevention, protection, cessation and regulation of tobacco consumption, as well as collaboration in the formulation of public policies and the implementation of tobacco control measures ^(^ ^5^ ^-^ ^6^ ^)^ . 

 In nursing research related to tobacco control, the most common nursing interventions are those aimed at smoking cessation. These include lifestyle assessment, identification of barriers to stopping smoking, assessment of smoking dependence, beliefs and values, rehabilitation of smokers, groups and training ^(^ ^7^ ^)^ . 

 The literature contains various instruments aimed at assessing predisposition to tobacco use and identifying the barriers that hinder the smoking cessation process, or even assessing satisfaction with counseling services ^(^ ^7^ ^-^ ^8^ ^)^ . 

 There are instruments that seek to understand the knowledge, beliefs, attitudes and lifestyle of individuals or specific populations in relation to cigarettes, such as The Knowledge, Attitudes, Behaviors and Organization questionnaire for Students, the Healthy Lifestyles Questionnaire (CEVS-II), Perinatal Tobacco Attitudes and Behaviors Survey (PTABS), Questionnaire on Smoking Urges (QSU) and Brief Tobacco Intervention (BTI) ^(^ ^9^ ^-^ ^13^ ^)^ . 

 However, the studies did not identify any instruments aimed at assessing nurses’ smoking cessation practices ^(^ ^7^ ^-^ ^8^ ^)^ . 

 Due to the scarcity of validated instruments to assess smoking cessation practices, a scale was developed to assess smoking cessation counseling practices applied in nurses’ clinical practice, called Smoking Cessation Counseling (SCC) ^(^ ^14^ ^)^ . Although there are instruments that assess different smoking-related constructs, only the SCC investigated the counseling construct ^(^ ^9^ ^-^ ^13^ ^)^ . 

 The SCC scale was developed by three nurses from the University of Maryland, Baltimore, in the United States of America (USA), who based the instrument on the guideline Helping smokers quit: A guide for nurses, published in 2005 by the United States (U.S) Department of Health and Human Services ^(^ ^14^ ^-^ ^15^ ^)^ . The guideline is based on the 5 A’s that are used by health professionals in the follow-up of smoking patients, which are Ask, Advise, Assess, Assist and Arrange ^(^ ^15^ ^)^ . 

 The scale was applied to 591 nurses from 23 rural hospitals in the eastern United States that treat patients with acute conditions such as heart failure, acute myocardial infarction and pneumonia. For these hospitals, smoking cessation counseling is considered a quality indicator for the aforementioned health conditions ^(^ ^14^ ^)^ . 

 The SCC consists of 26 items, the first 24 of which have a four-level Likert-type response format, indicating the frequency of nurse intervention in smoking cessation counseling in each item. The last two items assess, on a 10-point scale, the nurse’s self-perception of comfort in conducting smoking cessation counseling and referring smokers to smoking cessation resources ^(^ ^14^ ^)^ . 

 In the validation study of the original scale, various analyses were carried out. The total SCC score was calculated and correlated with the last two items, related to comfort in conducting counseling and referral in smoking cessation. In addition, a regression model was used to assess the relationship between the total SCC score and the competencies of comfort and referral in counseling, considering demographic variables such as education, gender, age and ethnicity. In addition, an exploratory factor analysis and reliability analysis was carried out on the first 24 items, which showed a Cronbach’s alpha coefficient of 0.955 ^(^ ^14^ ^)^ . 

 Smoking Cessation Counseling was first validated and adapted in China, showing construct validity and an internal consistency ranging from 0.56 to 0.79 for the instrument’s 24 items ^(^ ^16^ ^)^ . 

 The SCC was translated into Brazilian Portuguese and adapted for Brazilian culture, called Smoking Cessation Counseling - Brazilian Version (SCC-BV). This was done through translation, synthesis of the translations, back-translation, review of the back-translation by a committee of experts and a pre-test with 50 nurses, in which the internal consistency of the items in the instruments was assessed ^(^ ^17^ ^)^ . For the 24 items, evidence of reliability was found with a Cronbach’s alpha of 0.916, which indicates the instrument’s strong internal consistency. After translation and cross-cultural adaptation, other psychometric properties should be assessed in order to verify the validity of the Brazilian version of the SCC ^(^ ^17^ ^)^ . 

In view of the above, it is clear that obtaining evidence of the validity of the SCC-BV instrument will be useful for health services and nurses, as it will enable them to recognize the effectiveness and shortcomings of their smoking cessation intervention program.

The aim of this study was to assess the validity of the internal structure and reliability of the Brazilian version of the Smoking Cessation Counseling - Brazilian Version (SCC-BV).

## Method

### Study design

This is a psychometric study in which the properties of Smoking Cessation Counseling - Brazilian Version (SCC-BV) were evaluated by means of internal structure and reliability.

### Setting

The study was carried out in São Paulo - SP, Brazil.

### Period

Data was collected from January 2020 to November 2020.

### Population

The study population consisted of nurses from the state of São Paulo, who were members of the Sao Paulo regional office of the Reference Center for Alcohol, Tobacco and Other Drugs (CRATOD).

### Selection criteria

Nurses with specialist qualifications in cardiology, clinical medicine, oncology and public health or with at least two years’ experience in the field were included, as were professionals who work with smoking cessation counseling in a hospital or outpatient unit and those who had been certified by a reference center for alcohol, tobacco and other drugs to carry out smoking cessation counseling.

### Sample definition

 To calculate the sample, a sample of 10 participants was used for each item in the instrument ^(^ ^18^ ^-^ ^19^ ^)^ . The literature recommends that a sample should contain at least 100 subjects per factor measured ^(^ ^19^ ^)^ . In this sense, based on the 24 items of the SCC-BV, the minimum total number of participants required was 240 clinical practice nurses from the Sao Paulo state regional office. 

In view of the recommendations in the literature and in order to guarantee a return, the sample consisted of 250 nurses in clinical practice in the regional region of the state of Sao Paulo, in which we obtained 100% feedback from the nurses.

### Instrument used to collect information

 The Brazilian version of the Likert-type SCC scale includes 26 items and is divided into four domains, called domain 1 “Advanced Counseling” (items 7 to 16 and 20 to 24); domain 2 “Referral to Services” (items 17, 18 and 19); domain 3 “Basic Counseling” (items 3, 4, 5 and 6); domain 4 “Standard Care” (items 1 and 2). The first 24 questions have a four-level response format, indicating the frequency of nurse intervention in smoking cessation counseling with scores ranging from 1 to 4, indicating 1 “never”, 2 “less than half the time”, 3 “more than half the time” and 4 “all the time”. The last two assess the nurse’s self-perception in relation to comfort in conducting smoking cessation counseling, and referral to available resources, assigning values from 1 to 10, with 1 indicating “not at all comfortable” and 10 “very comfortable”. There is also a field for comments, if necessary ^(^ ^14^ ^)^ . 

 The SCC score can range from 24 to 96. A score of 24 is the lowest score, when all the answers were 1 (Never), while a score of 96 is the best counseling, when all the answers were 4 (All the time). The score from 24 to 96 indicates the sum of the frequency of nurse intervention in smoking cessation counseling for the first 24 items on the scale ^(^ ^14^ ^)^ . 

### Data collection

Participants received an invitation to take part by email, containing an electronic form with an Informed Consent Form (ICF), a sociodemographic questionnaire and the Brazilian version of the SCC scale. The deadline for returning the scale was two weeks. If it wasn’t returned, the scale would be sent again and they would have to wait another two weeks.

### Data treatment and analysis

Initially, the data was analyzed using descriptive statistics, in which the qualitative variables were described using frequencies and percentages, and the quantitative variables using measures of position (mean and median) and dispersion (standard deviation and quartiles). A significance level of 5% was considered for all analyses.

 In order to assess the psychometric properties, this study analyzed the structural validity of the SCC-BV instrument by assessing dimensionality and reliability. To assess the dimensionality of the SCC-BV, 2 ^nd^ order confirmatory factor analysis was carried out, using structural equation models with Partial Least Squares (PLS) as the estimation method. Analysis of the factor model was carried out in two stages: convergent validity and discriminant validity. 

 In order to analyze the convergent validity of the factor model, the AVE (Average Variance Extracted) results for each of the model’s factors were initially evaluated. This measure assesses the proportion of the variance of the items that is explained by the factor to which they belong. AVE values greater than 0.5 indicate that the model converges to a satisfactory result ^(^ ^20^ ^)^ . 

 Discriminant validity was initially assessed using the Fornell-Larcker criterion ^(^ ^21^ ^)^ . This method compares the square roots of the AVEs with the correlation values between the factors. Another criterion used to assess discriminant validity was the analysis of cross loadings. In this case, it was observed whether the factor loading of a given item was higher in the factor in which it was initially allocated than in the other factors of the model. 

 Cronbach’s alpha coefficient and composite reliability were calculated in order to assess the instrument’s internal consistency. Values above 0.7 were considered satisfactory ^(^ ^20^ ^,^ ^22^ ^)^ . 

### Ethical aspects

 The research project was approved by the Research Ethics Committee of the *Escola Paulista de Enfermagem* from *Universidade Federal de São Paulo* , São Paulo, under the Certificate of Submission for Ethical Appraisal (CAAE) 04737012.7.0000.5505. Authorization to validate the SCC was granted by the instrument’s author and all study participants signed the Informed Consent Form (ICF). 

## Results

 A total of 250 nurses took part in the study, with an average age of 33 and an average length of experience of 7.11 years in the field. Most of the participants were women, worked 8-hour shifts, specialized in Public Health and had professional experience in outpatient sectors ( Table 1 ). 

**Table 1:** - Sociodemographic and work characterization of the nurses participating in the study (n*=250). São Paulo, SP, Brazil, 2020

**Variable**	N (Mean)*
**Average age (SD ^†^ )**	33 (3,89)
**Average length of experience (SD ^†^ )**	7,11 (2,63)
**Gender (% ^‡^ )**	
Male	37 (14,8)
Female	213 (85,2)
**Specialization (% ^‡^ )**	
Cardiology	50 (20)
Clinical Medicine	15 (6,0)
Postgraduate *Stricto Sensu* (Doctorate)	6 (2,4)
Postgraduate *Lato Sensu* (Master’s)	13 (5,2)
Oncology	24 (9,6)
Public health	142 (56,8)
**Professional experience (% ^‡^ )**	
Outpatient	151 (60,4)
Hospital	99 (39,6)
**Working hours (% ^‡^ )**	
6 hours	63 (25,2)
8 hours	151 (60,4)
12 hours	36 (14,4)
**Area of activity (% ^‡^ )**	
Outpatient medical care (AMA ^§^ )	32 (12,8)
Psychosocial care center (CAPS ^||^ )	27 (10,8)
Infirmary	54 (21,6)
Health center	92 (36,8)
Coronary Care Unit (CCU ^¶^ )	21 (8,4)
Intensive Care Unit (ICU**)	24 (9,6)

* Mean

^†^ SD = Standard Deviation

^‡^ % = Percentage

^§^ AMA = Ambulatory Medical Care

^||^ CAPS = Psychosocial Care Center

^¶^ CCU = Coronary Unit

** ICU = Intensive Care Unit

 Regarding the frequency of responses to the SCC-BV, Table 2 shows that the frequency of use of counseling interventions for smoking cessation was reported as “all the time” by most nurses for items 1, 2, 3 and 4 and “more than half the time” for items 5 to 24. 

Items 6, 7, 8, 14, 17, 19, 20, 21, 23 and 24 were evaluated by the nurses as “less than half the time” and with frequencies between 15 and 35% of the nurses, while in relation to the frequency of use “never”, there was a frequency of response between 0 and 8% of the nurses, with the exception of items 3 and 5 which did not present the frequency of use “never”.

In relation to item 25, which is related to the level of comfort in conducting smoking cessation counseling, a mean score of 7.2 (SD = 1.55) was obtained and comfort in referring patients for smoking cessation counseling in item 26 with a mean score of 6.8 (SD = 1.58).

**Table 2:** - Frequency of responses to the items in the Brazilian version of Smoking Cessation Counseling (n*=250). São Paulo, SP, Brazil, 2020

	n(%)*			
	1	2	3	4
SCC1 ^†^ - I assess my patient’s tobacco use.	1(0,40)	26(10,40)	77(30,80)	146(58,40)
SCC2 ^†^ – I record my patient’s tobacco use.	3(1,20)	46(18,40)	58(23,20)	143(57,20)
SCC3 ^†^ - I advise tobacco users to stop smoking.	_	40(16,00)	65(26,00)	145(58,00)
SCC4 ^†^ – I ask tobacco users if they are willing to quit at this time.	1(0,40)	44(17,60)	61(24,40)	144(57,60)
SCC5 ^†^ – If tobacco users are willing to quit, I provide resources and assistance.	_	14(5,60)	159(63,60)	77(30,80)
SCC6 ^†^ – If tobacco users are not willing to quit, I provide resources and help identify barriers to quitting.	5(2,00)	42(16,80)	141(56,40)	62(24,80)
SCC7 ^†^ – I advise smokers to set a quit date.	5(2,00)	42(16,80)	149(59,60)	54(21,60)
SCC8 ^†^ – I advise smokers to get support from family, friends and coworkers.	7(2,80)	50(20,00)	138(55,20)	55(22,00)
SCC9 ^†^ – I review past attempts to quit smoking - what helped, what led to relapses.	6(2,40)	29(11,60)	171(68,40)	44(17,60)
SCC10 ^†^ – I help the patient anticipate challenges, particularly during key critical weeks.	1(0,40)	28(11,20)	161(64,40)	60(24,00)
SCC11 ^†^ – I help the patient anticipate nicotine withdrawal.	3(1,20)	21(8,40)	174(69,60)	52(20,80)
SCC12 ^†^ – I identify reasons for quitting and the benefits of quitting.	2(0,80)	28(11,20)	152(60,80)	68(27,20)
SCC13 ^†^ – I advise patients that total abstinence is essential - not even a puff.	3(1,20)	36(14,40)	150(60,00)	61(24,40)
SCC14 ^†^ – I counsel patients that alcohol consumption is strongly associated with relapse.	5(2,00)	43(17,20)	150(60,00)	52(20,80)
SCC15 ^†^ – I advise patients that having other smokers in the house makes it difficult to quit successfully.	3(1,20)	22(8,80)	193(77,20)	32(12,80)
SCC16 ^†^ – I recommend the use of patches, chewing gum or nicotine lozenges, or get a prescription for a nasal spray, inhaler or Bupropion, unless contraindicated.	4(1,60)	31(14,40)	193(77,20)	22(8,80)
SCC17 ^†^ – I provide the number of Health Dial 136.	13(5,20)	72(28,80)	156(62,40)	9(3,60)
SCC18 ^†^ – I refer the patient to the online resources of the Ministry of Health/National Tobacco Control Program.	9(3,60)	92(36,80)	132(52,80)	17(6,80)
SCC19 ^†^ – I refer the patient to the online resources for “Step by Step to Quit Smoking”.	11(4,40)	89(35,60)	128(51,20)	22(8,80)
SCC20 ^†^ – I use cessation materials that are appropriate for age, culture, language, education and pregnancy status.	3(1,20)	86(34,40)	154(61,60)	7(2,80)
SCC21 ^†^ – I provide information for follow-up visits with the patient’s doctor, nurse/multidisciplinary team.	4(1,60)	38(15,20)	121(48,40)	87(34,80)
SCC22 ^†^ – I advise patients that if they relapse, they should repeat their quit attempts - it’s part of the quitting process.	2(0,80)	32(12,80)	188(75,20)	28(11,20)
SCC23 ^†^ – I advise patients that if relapses occur, they should review the circumstances and learn from the experiences.	4(1,60)	49(19,60)	186(74,40)	11(4,40)
SCC24 ^†^ – I advise patients that if relapses occur, they should re-evaluate the use and problems of pharmacotherapy.	11(4,40)	47(18,80)	173(69,20)	19(7,60)

* n(%) = Frequency of responses to items

^†^ SCC = Scale items

 The evaluation of the instrument’s internal structure (confirmatory factor analysis) was based on the structure of the domains proposed in the original study of the adapted instrument ^(^ ^14^ ^,^ ^17^ ^)^ . 

Initially, the model’s convergent validity was assessed, and AVE values of less than 0.5 were identified in domain 1. The items with the lowest factor loading values were then excluded until a satisfactory AVE value was obtained. In this process, seven items were excluded from domain 1 (items 15, 16, 20, 21, 22, 23 and 24), achieving an AVE value borderline to the established value. The other domains had AVE values above 0.50, as shown in Table 3.

**Table 3:** - AVE*, composite reliability and Cronbach’s alpha of the initial and final model of the SCC ^†^ instrument. São Paulo, SP, Brazil, 2020

	**Initial instrument model**		**Final instrument model**	
	**Domain**	Value	**Domain**	Valor
AVE*	**D1** ^‡^	0,29	**D1** ^‡^	0,47
	**D2** ^§^	0,52	**D2** ^§^	0,52
	**D3** ^||^	0,50	**D3** ^||^	0,64
	**D4** ^¶^	0,76	**D4** ^|¶^	0,76
**Composite reliability**	**D1** ^‡^	0,84	**D1** ^‡^	0,88
	**D2** ^§^	0,76	**D2** ^§^	0,76
	**D3** ^||^	0,79	**D3** ^||^	0,84
	**D4** ^¶^	0,86	**D4** ^¶^	0,86
**Internal consistency**	**D1** ^‡^	0,80	**D1** ^‡^	0,84
	**D2** ^§^	0,53	**D2** ^§^	0,53
	**D3** ^||^	0,65	**D3** ^||^	0,71
	**D4** ^¶^	0,69	**D4** ^¶^	0,69
	**Global Score**	0,87	**Global Score**	0,86

* AVE = Average Variance Extracted

^†^ SCC = Smoking Cessation Counseling

^‡^ D1 = Domain 1

^§^ D2 = Domain 2

^||^ D3 = Domain 3

^¶^ D4 = Domain 4

**Figure 1:**
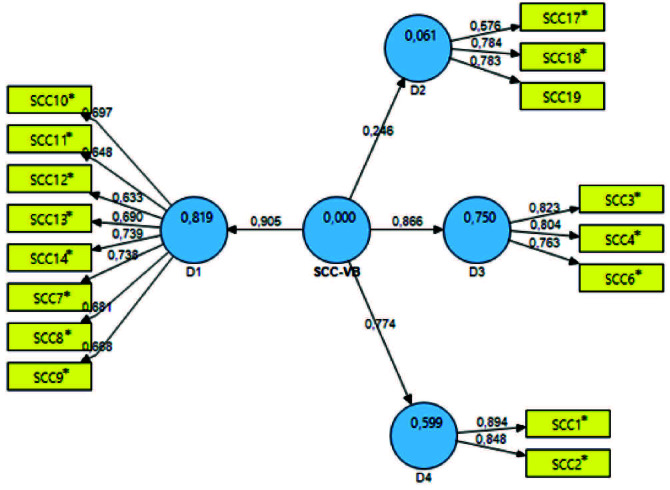
- Final model of the SCC-BV. São Paulo, SP, Brazil, 2020

Source: Smart PLS 2.0

^⁎^ SCC= Scale items

It should be noted that the exclusion of items also took into account the theoretical/conceptual relationship of the items with the construct and the domains.

Subsequently, the discriminant validity of the model was assessed using the Fornell-Larcker criterion and the cross-loadings of the items to determine the final structural model. At this stage it was necessary to exclude item 5 from domain 3, as this item had a higher load in domain 1.

 After these exclusions, the final version of the instrument was obtained, as shown in Figure 1 , consisting of 4 domains, called domain 1 “Advanced Counseling” (items 7, 8, 9, 10, 11, 12, 13 and 14); domain 2 “Referral to Services” (items 17, 18 and 19); domain 3 “Basic Counseling” (items 3, 4 and 6); domain 4 “Standard Care” (items 1 and 2). 

In the composite reliability analysis, values higher than 0.70 were obtained in all domains. However, in the analysis of internal consistency using the Cronbach’s alpha coefficient, values lower than 0.70 were obtained in the Referral to Services and Standard Care domains, although a coefficient of 0.86 was obtained for the overall instrument, as shown in Table 3.

## Discussion

 Among the nurses’ characteristics, the majority were women, with an average age of 33 and an average length of experience of 7.11 years in the field. Most of them worked eight-hour shifts, specialized in nursing and had professional experience in outpatient settings. This study presented similar characteristics when compared to the original study, except for the place of work, due to the classification of health services in Brazil, which allows for greater monitoring of tobacco users in the outpatient setting ^(^ ^14^ ^,^ ^23^ ^)^ . 

The aspects of smoking counseling reported by the nurses with the highest frequency of use “all the time” and “more than half the time” were: basic counseling, standard care and referral to services. These items consist of assessment interventions, documentation of tobacco use, counseling users about their willingness to quit smoking and identifying possible barriers in the process of quitting.

The items of providing appropriate resources for age, culture, language, pregnancy and care, referral to resources such as the Ministry of Health’s Tobacco Control Program and Health Dial 136, as well as pharmacotherapy, showed less frequency of use classified as “less than half the time” and “never”.

 In the literature, there is research that has studied the approach of nurses to tobacco users with the aim of understanding the experience of these professionals in conducting smoking cessation counseling. The studies show that nurses have difficulties in identifying appropriate resources for each audience, referring and applying the resources of the Tobacco Control Program, as well as providing pharmacotherapy for smokers assisted in primary health care centers ^(^ ^6^ ^,^ ^14^ ^,^ ^24^ ^-^ ^26^ ^)^ . 

 The research cited above shows that these difficulties are explained by the lack of training for professionals, high turnover of professionals in the teams, lack of professionals from other areas and lack of medicines in the health services ^(^ ^6^ ^,^ ^14^ ^,^ ^24^ ^-^ ^26^ ^)^ . The nurses also report the need to increase the frequency of training provided by the Ministry of Health, which focuses on the individual approach to smokers through exclusive nursing consultations with smoking patients ^(^ ^6^ ^)^ . 

 According to the nurses’ statements, the individual approach to tobacco-dependent users by nurses in Primary Health Care is unsystematic. Although the unsystematic approach to encourage smoking cessation is a relevant sensitizing strategy in nurses’ clinical practice, it does not provide organized and structured nursing care. When approaching smokers, nurses need to use the Nursing Process (NP) as a working tool to ensure good care practices for this clientele. By associating the NP with the individual and collective approach, nurses can maximize the effect of interventions and broaden the range of activities aimed at smokers in Primary Health Care ^(^ ^6^ ^)^ . 

 The studies on the original version of the SCC presented as the most frequent aspects of tobacco treatment the items of providing resources and assistance, tobacco assessment and documentation, counseling and asking tobacco users if they are willing to quit. The practices most frequently reported as never having been carried out were referring patients to get more information for smoking cessation treatment, health research, a free stop smoking disk tool, pharmacotherapy and setting a quit date ^(^ ^14^ ^,^ ^27^ ^)^ . Therefore, the similarities in the adoption of strategies involving the provision of resources, assessment, counseling and motivation for smoking cessation were evident, and practices that need greater attention and implementation in this context were pointed out, in relation to the international results of the SCC. 

 For the items on comfort skills in conducting and referring smoking cessation counseling, the nurses rated their skills as average, on a scale of 0 to 10 points, 7.2 and 6.8. These values corroborate the data identified in studies on the original version of the SCC ^(^ ^14^ ^,^ ^27^ ^)^ , which showed that the aforementioned skills also had average reports. 

 The structural validity of the instrument consisted of assessing dimensionality by means of confirmatory factor analysis of the domain structure, which was proposed in the original study of the adapted instrument. Both the studies on the original version of the SCC and the validation study of the instrument in China assessed the structural validation of the SCC by means of exploratory factor analysis, but both reported the need to carry out confirmatory factor analysis studies in order to obtain more psychometric evidence ^(^ ^14^ ^,^ ^16^ ^,^ ^27^ ^)^ . 

 In order to replicate and validate instruments for other cultures, it is necessary to translate the instrument, and during translation, one or more questions can lose their meaning, which can lead to a change in the interviewees’ understanding. In this case, confirmatory factor analysis plays the role of comparing whether the same questions in the questionnaire continue to form the same constructs in the study. This is because, due to translation, one or more variables may no longer be correlated with the other variables in their respective constructs, and in some cases they may even be going in the opposite direction ^(^ ^28^ ^)^ . 

 The original SCC study used exploratory factor analysis for structural validation, allowing the main components or sub-scales to be identified, examining redundancies between items. The analyses indicated that all the items correlated well with each other, maintaining the 24 items. In the analysis of the internal consistency of the 24 items, a Cronbach’s alpha coefficient of 0.9 was identified in the original study, estimating high levels of reliability ^(^ ^14^ ^)^ . 

 In this study, 7 items were excluded from domain 1 in an attempt to increase the AVE value in these domains. These seven excluded items dealt with relapse counseling, barriers that hinder the process of quitting smoking, knowledge and provision of pharmacotherapy, multidisciplinary assistance, appropriate materials and resources. These excluded items were reported by nurses less frequently during counseling, demonstrating weaknesses in training and a lack of resources in health services. In view of this, the exclusions were justified during the convergent validity analysis process. The application of the items mentioned requires continuous training and the provision of resources by government bodies, in order to enable nurses to understand all the aspects, concepts, treatments and approaches related to the care of smoking patients ^(^ ^6^ ^,^ ^24^ ^-^ ^26^ ^)^ . Brazilian studies show that nurses report a shortage of training and resources to conduct smoking cessation counseling ^(^ ^6^ ^,^ ^24^ ^)^ , which justifies the behavior of the data and the AVE values for the aforementioned items. 

The excluded items (15, 20, 22, 23 and 24) are similar to the items from domains 1 and 2 kept in the instrument, such as items SCC8 - I advise smokers to get support from family, friends and coworkers; SCC18 - I refer the patient to online resources from the Ministry of Health/National Tobacco Control Program; SCC19 - I refer the patient to online resources for “Step by Step to Quit Smoking”; SCC9 - I review past attempts to quit smoking - what helped, what led to relapses; SCC10 - I help the patient anticipate challenges, particularly during key critical weeks; SCC13 - I counsel patients that total abstinence is essential - not even one puff; SCC14 - I counsel patients that alcohol consumption is strongly associated with relapses, respectively, which lessens the impact of excluding the aforementioned items.

 The low factor loadings of item 16, which refers to recommending smoking cessation methods such as patches, chewing gums, nicotine lozenges or prescribing other treatments, and item 21, which refers to providing information during joint visits with health professionals, can be attributed to cultural differences and the level of nurse autonomy between the country where the scale originated ^(^ ^14^ ^)^ and Brazil. 

After excluding the aforementioned items, domain 1 had an AVE value bordering on the established value, and the authors decided to maintain this value, without excluding any other items, since this result is very close to the minimum required.

During the discriminant validity of the model, item 5 (“If tobacco users are willing to quit, I provide resources and assistance”) of domain 3 correlated better with domain 1. This is due to the fact that domain 1 includes the advanced actions applied during counseling, such as advising tobacco users to anticipate nicotine withdrawal, identifying the benefits of quitting, relapses, factors that hinder the process of quitting smoking, pharmacotherapy and referral to resources. Therefore, item 5 was excluded. However, it is important to note that the actions previously addressed in this item were incorporated into items 18 and 19, which were kept in the final version of the instrument (“Referral of the patient to online resources of the Ministry of Health/National Tobacco Control Program” and “Step by Step to Quit Smoking”).

 The composite reliability of the SCC-BV instrument was higher than 0.70 in all domains and this calculation is based on the possibility of variation in factor loadings or weights, making it a more robust indicator of accuracy when compared to the Cronbach’s alpha coefficient ^(^ ^29^ ^)^ . 

 In the analysis of consistency using the Cronbach’s alpha coefficient, we obtained values of between 0.53 and 0.80 in the final version, depending on the domain assessed. However, in the overall analysis of the instrument, we achieved very close values (Cronbach’s alpha 0.86) to the original version ^(^ ^14^ ^)^ (Cronbach’s alpha 0.90). Some domains showed Cronbach’s alpha values lower than 0.70, but all showed composite reliability values higher than this value. 

 In the psychometric evaluation of the Chinese version of the instrument, the value ranged from 0.56 to 0.79, when analyzing the structure of the instrument with four factors, values very close to those achieved in this study, however when the Chinese study analyzed the instrument with a three-factor structure, a Cronbach’s alpha coefficient of 0.96 was obtained for the overall score and 0.94 for the advanced counseling domain, 0.93 for the basic counseling domain and 0.80 for the referral to services domain, this when compared to the original scale ^(^ ^16^ ^)^ . 

One of the reasons for the reduction in the Cronbach’s alpha coefficient in the domains related to referral to services may be related to the low frequency of referrals to support services made by the nurses interviewed. As for the Basic Care domain, it may be related to the low number of items in it, and it is important to note that the value obtained in this domain was borderline to the established minimum.

After these exclusions, a final version of the instrument was obtained containing 16 items, distributed in 4 domains, with satisfactory AVE values, composite reliability and adequate results according to the Fornell-Larcker criterion and the cross-load analysis, in which the SCC-BV presented a structural model convergent to its construct and a structural model in which the items in each domain correlate.

This study has limitations, since the stability and validity of known groups or contrasted groups were not analyzed. These psychometric properties will be studied in a future study in order to accumulate more evidence of validity and reliability.

## Conclusion

The structural validation procedures for the SCC-BV were successfully completed in accordance with the recommendations in the literature, resulting in an instrument made up of 16 items distributed over 4 domains: standard counseling, basic counseling, referral to services and advanced counseling. Composite reliability values were between 0.76 and 0.88, and the overall internal consistency of the instrument was 0.86.

This study contributes to the national literature, as it provides a tool with adequate evidence of validity for evaluating smoking cessation counseling from the perspective of nurses working in health services.
